# Effect of Sea-Buckthorn (*Hippophaë rhamnoides* L.) Pulp Oil Consumption on Fatty Acids and Vitamin A and E Accumulation in Adipose Tissue and Liver of Rats

**DOI:** 10.1007/s11130-017-0610-9

**Published:** 2017-05-02

**Authors:** Sylwester Czaplicki, Dorota Ogrodowska, Ryszard Zadernowski, Iwona Konopka

**Affiliations:** 1 Chair of Plant Food Chemistry and Processing, Faculty of Food Sciences, University of Warmia and Mazury, Plac Cieszyński 1, 10-726 Olsztyn, Poland; 2 Department of Agriculture and Economics, The Academy of Agrobusiness in Łomża, Studencka 19 Str, 18-402 Łomża, Poland

**Keywords:** Sea-buckthorn oil, Fatty acids, α-tocopherol, β-carotene, Retinol, Animal study

## Abstract

An *in vivo* experiment was conducted to determine the effect of sea-buckthorn pulp oil feeding on the fatty acid composition of liver and adipose tissue of Wistar rats and the liver accumulation of retinol, its esters and α-tocopherol. For a period of 28 days, rats were given a modified casein diet (AIN-93) in which sea-buckthorn pulp oil, soybean oil and pork lard were used as sources of fat. Compared to the other fat sources, sea-buckthorn pulp oil was the most abundant in C16 fatty acids, carotenoids (mainly β-carotene) and tocopherols (mainly α-tocopherol). Its consumption was reflected in an increased share of palmitoleic acid in adipose tissue and the liver and an increased level of retinol in liver tissues (this was not observed for its esters). Although the type of fat did not have a significant effect on the average content of α-tocopherol in the liver, the variation of saturation of this tissue with α-tocopherol was the lowest when rats were fed a diet containing sea-buckthorn oil. This experiment indicates the possibility of affecting adipose tissue and liver by a diet.

## Introduction

Plant fats differ in the composition of fatty acids and accompanied lipid-soluble compounds [1]. Canola, soybean, sunflower and olive oils are a basic dietary source of polyunsaturated fatty acids (PUFA), especially those of the C18-chain length, which are precursors of essential long-chain fatty acids (*e.g.*, arachidonic (ARA), docosahexaenoic (DHA), eicosapentaenoic (EPA), etc.) [2]. Other basic market fats, such as coconut and palm kernel oils, are a source of medium chain saturated acids broadly utilized in confectionery products, frying fats, chocolate, etc. In contrast, oils abundant in rare fatty acids, such as ω-3, ω-7, selected ω-9 and conjugated are often components of nutraceutical supplements or functional food.

Fatty acids are primarily energy sources and membrane constituents for the human body, but they have also many other biological activities related to chain length, saturation and number and the position of double bonds [3]. For example, 18 chain length ω-3 and ω-6 acids may be converted into signalling molecules such as prostaglandins, leukotrienes and thromboxanes [4]. Similarly, rare in the typical diet palmitoleic acid (ω-7) probably plays an important role in the regulation of hepatic versus adipocyte lipogenesis [3, 5]. Consuming food rich in this acid may beneficially affect both energy, homeostasis and metabolic health [5], for example, trans-palmitoleic acid in plasma phospholipids is strongly inversely associated with incident T2 diabetes mellitus [6]. This acid also reduces appetite and may help to combat other diseases, although the mechanism of its action has not been fully elucidated [7, 8].

Although various food groups (*e.g.*, fish) can deliver a proper quantity and quality of fatty acids, the unique trait and advantage of plant oils is the simultaneous supply of numerous regulatory and protective compounds such as sterols, tocols, carotenoids, and lipophilic phenolics [9, 10]. Non-refined oils (such as cold-pressed oils from pumpkin, linseed, evening primrose and sea-buckthorn) are great examples of matrices rich in lipid-soluble bioactive substances [1]. Oil made from the pulp of sea-buckthorn is special in this regard because of its high level of carotenoids and tocols [11, 12]. Some sea-buckthorn genotypes could deposit up to ca. 530 mg of carotenoids and up to ca. 200 mg of tocols in 100 g of pulp oil, accompanied by a ca. 40% share of palmitoleic acid (C16:1, ω-7) [13]. Since the data [1] shows that sea-buckthorn pulp oil, compared to other biooils, is one of the richest sources of these compounds in nature, it is not surprising that the market offer of functional food products and supplements with this oil is still growing. Although its pulp oil concentration is relatively low (approx. 3–5% of wet moisture), this morphological part may constitute up to 90% of total fruit and, in this regard, can be regarded as an efficient source of valuable oil.

Even if a human meal contains a nutritionally-appropriate composition of compounds, the physiological utilization of individual components depends on its solubilisation from the food matrix, uptake from the small intestine and subsequent transformation into chylomicrons and lipoproteins [14, 15]. Plasma delivery of lipid-origin compounds (such as α-tocopherol) is also affected by body/tissue saturation with this compound [16]. Generally, the absorbed fatty acids and low molecular lipophilic compounds are utilized at once or deposited as storage material, mainly in adipose tissue and the liver.

The main aim of this study was to determine if the consumption of sea-buckthorn pulp oil (compared to soybean oil and pork lard as a control diet lipids, which are highly differentiated in fatty acids and phytochemicals composition) affects the fatty acid profile of liver and adipose tissue of Wistar rats fed a modified casein diet (AIN-93). The second aim was to test if an increased concentration of carotenoids and tocopherols in sea-buckthorn pulp oil may affect retinol and its esters and the α-tocopherol content in the liver of the tested animals.

## Materials and Methods

### Study Material

Pork lard and soybean (*Glycine max* L.) oil were bought as foodstuffs at a retail outlet. Sea-buckthorn pulp oil was obtained from a laboratory. For this purpose, the fruit was disintegrated (MPW-324 homogeniser, Mechanika Precyzyjna, Warsaw, Poland) and then pressed with a hydraulic press (DEMA Vertriebs-GmbH, Im Tobel, Germany. The obtained juice was centrifuged at ca. 13,000 x g (5810R Eppendorf AG centrifuge, Hamburg, Germany) to separate liberated oil. The oil was collected, washed and purified by repeated centrifuging. The used fats were analysed for the content of carotenoids, tocopherols and fatty acid composition.

### *In Vivo* Nutritional Test

The experiment was carried out at the Department of Biological Function of Food, Institute of Animal Reproduction and Food Research of Polish Academy of Sciences, Olsztyn, Poland. All procedures and experiments complied with the guidelines and were approved by the Local Ethics Commission of the University of Warmia and Mazury in Olsztyn, Poland. Male rats (aged eight weeks) of the Wistar strain were fed for 28 days on a modified casein diet for laboratory rodents (AIN-93) composed of: fructose (30%), maize starch (29.5%), casein (20%), fat (10%), cellulose (5%), cholesterol (0.5%), mineral mix (3.5%), vitamin mix (1%), DL-methionine (0.3%) and cholic acid (0.2%).

A total of 24 animals were divided into three distinct groups (each of eight rats) fed on pork lard, soybean oil and sea-buckthorn oil as a fat source. Animals were fed *ad libitum* with continuous access to distilled water and maintained under standard conditions (temperature of 21–22 °C; relative humidity of 50–70% with intensive room ventilation (15×/h); 12-h lighting regimen). At the termination of the experiment, the rats were anesthetized with sodium pentobarbital according to the recommendations for the euthanasia of laboratory animals (50 mg/kg body weight) and material for analyses was taken. Liver and adipose tissue were homogenised and frozen until the time of the analyses of the neutral lipid fatty acid profile (adipose tissue and liver) and the content of retinol and its esters and tocopherols (only liver). The remaining animal tissues and blood were used in other experiments.

### Analytical Procedures

#### Isolation of Adipose Tissue and Liver fat

Extraction was conducted according to the method of Hosotani and Kitagawa [17]. A sample (0.2 g) of ground tissue was weighed within an accuracy of 0.001 g and homogenised with 0.2 ml of distilled water. The samples were then precipitated with 0.8 mL of ethanol (96%) and lipophilic compounds were extracted using 1 mL of hexane in triplicate. The solvent was evaporated at 30 °C at a reduced pressure in an Eppendorf rotary concentrator (Eppendorf AG, Hamburg, Germany). Extracts were dissolved in hexane, centrifuged (5 min, ca. 13,000 x g) and taken for an assay of retinol, retinol esters, tocopherols and determination of the fatty acid profile.

#### Analytical Methods

Fatty acid methyl esters were analysed by gas chromatography with a GC-MS QP2010 PLUS (Shimadzu, Japan) system according to the method described by Czaplicki et al. [11]. Briefly, fatty acid methyl esters were separated on a BPX70 (25 m × 0.22 mm × 0.25 μm) capillary column (SGE Analytical Science, Victoria, Australia) with helium as the carrier gas at a flow rate of 0.9 mL/min. The column temperature was programmed as follows: a subsequent increase from 150 to 180 °C at the rate of 10 °C/min, to 185 °C at the rate of 1.5 °C/min, to 250 °C at the rate of 30 °C/min and a 10 min hold. The interface temperature of GC-MS was set at 240 °C. The temperature of the ion source was 240 °C and the electron energy was 70 eV. The total ion current (TIC) mode was in the 50–500 m/z range.

Carotenoids were analysed with a RP-HPLC technique according to Czaplicki et al. [11]. Briefly, the analysis was carried out using a 1200 series liquid chromatograph manufactured by Agilent Technologies (Palo Alto, CA, USA), equipped with a diode array detector (DAD) from the same manufacturer. Separation was performed at 30 °C on a YMC-C30 250 × 4.6 mm, 5 μm column (YMC-Europe GmbH, Germany). A methanol- methyl tert-butyl ether (MTBE) gradient was programmed as it is presented in Table 1.

**Table 1:** Parameters of the HPLC gradient used to separate carotenoids

Time (min)	Flow rate (mL/min)	Methanol (%)	MTBE (%)
0	1	95	5
5	1	95	5
25	1.25	72	28
33	1.25	5	95
40	1	95	5
60	1	95	5

The absorbance was measured at the wavelength of 450 nm for carotenoids and 325 nm for retinol and retinol esters. Compounds were identified based on retention times of commercially available standards (Sigma-Aldrich Sp. z o.o., Poznań, Poland).

The analysis of tocopherols was carried out by the NP-HPLC technique according to Czaplicki et al. [11]. Briefly, the analysis was performed using a 1200 series liquid chromatograph manufactured by Agilent Technologies (Palo Alto, CA, USA), equipped with a fluorescence detector from the same manufacturer. The separation was done on a Merck LiChrospher Si 60 column, 250 mm × 4 mm, 5 μm. A 0.7% isopropanol solution in hexane at a 1 mL/min flow rate was used as the mobile phase. The fluorescence detector was set at 296 nm for excitation and 330 nm for emission. Peaks were identified on the basis of retention times determined for α-, β-, γ- and δ-tocopherol standards (Merck, Darmstadt, Germany) separately, and their content was calculated using external calibration curves.

The statistical analyses were carried out with the STATISTICA 10.0 PL package (StatSoft Polska sp. z o.o., Kraków, Poland). The average values were compared by means of a one-way analysis of variance with Duncan’s test at *p* = 0.05 as the critical level of significance.

## Results and Discussion

### Fatty Acid Composition, Carotenoids and Tocopherols in Fats of Experimental Diets

The fatty acid profile of experimental diets is shown in Table 2. Sea-buckthorn oil differed from other fats by its extremely high content of C16 fatty acids, in which the share of palmitic acid was 36.3% and palmitoleic acid was 41.0%. Sea-buckthorn fruit oil may be considered as one of the richest source of palmitoleic acid [18, 19]. The total share of PUFAs in this oil was only 13.4%, with the prevalence of linoleic acid (ca. 90%). Sea-buckthorn oil was similar in the share of saturated, mono and polyunsaturated acids to pork lard. The composition of soybean oil was quite different, with the prevalence of linoleic (52.5%) and oleic (25.5%) acids. The total share of PUFAs was 58.4%, *i.e.*, above 4- and 9-fold higher than in sea-buckthorn oil and in lard. The composition of used oils was consistent with previous data [11, 18, 20, 21].

**Table 2:** Fatty acids composition (%) and bioactive compounds content in fats used in experiment (mg/100 g)

Fatty acids	Pork lard	Soybean oil	Sea-buckthorn oil
myristic C_14:0_	1.89 ± 0.05	trace	trace
palmitic C_16:0_	27.30 ± 0.42	12.90 ± 0.62	36.31 ± 0.01
palmitoleic C_16:1 ω7_	2.88 ± 0.02	nd	40.97 ± 0.04
stearic C_18:0_	15.81 ± 0.00	3.12 ± 0.02	0.55 ± 0.01
oleic C_18:1 ω 9_	45.94 ± 0.28	25.54 ± 0.19	8.77 ± 0.11
linoleic C_18:2 ω6_	5.50 ± 0.20	52.49 ± 0.71	12.12 ± 0.13
α-linolenic C_18:3 ω3_	0.42 ± 0.04	5.95 ± 0.09	1.28 ± 0.04
γ-linolenic C_18:3 ω6_	0.26 ± 0.05	nd	nd
SFA	45.00	16.02	36,86
MUFA	48.82	25.54	49.74
PUFA	6.18	58.44	13.40
ω3/ ω6 ratio	0.07	0.11	0.11
lutein	nd	trace	3.24 ± 0.49
zeaxanthin	nd	nd	1.65 ± 0.14
β-cryptoxanthin	nd	nd	3.94 0.36
α-carotene	nd	nd	6.90 0.74
β-carotene	trace	trace	118.36 ± 9.58
9-cis-β-carotene	trace	trace	15.56 ± 1.12
unidentified	nd	nd	56.39 ± 5.01
total carotenoids	trace	trace	206.04 ± 15.63
α-tocopherol	3.06 ± 0.41	15.01 ± 0.30	144.14 ± 4.10
β-tocopherol	nd	nd	3.98 ± 0.23
γ-tocopherol	nd	53.35 ± 5.32	4.63 ± 0.32
δ-tocopherol	nd	31.03 ± 1.61	0.75 ± 0.00
total tocopherols	3.06 ± 0.41	99.39 ± 5.42	153.50 ± 4.25

nd – not detected

Characteristic of carotenoids and tocols of experimental diets lipids is shown in Table 2. Sea-buckthorn oil contained considerable amounts of carotenoids (206.0 mg/100 g). This value lies within the limits given by other studies, although composition of this oil is generally highly variable in relation to cultivar, degree of fruit ripeness, origin of oil (from seed or fruit pulp) and method of extraction [22, 23]. Carotenoid-like compounds of this oil were classified as carotenes (ca. 68% of total fraction), xanthophylls (ca. 4%) and unidentified compounds (28%). All-trans β-carotene prevailed (ca. 84%) among carotenes. The presence of α- and 9-cis-β-carotene was also noted. Among xanthophylls, compounds such as lutein, zeaxanthin and β-cryptoxanthin were identified. Both comparative fat sources contained only traces of carotenoids.

In contrast, tocopherols were present in all tested fat matrices. The highest content of tocopherols (153.5 mg/100 g) was determined in sea buckthorn oil, and α-tocopherol accounted for 94% of the total. Soybean oil was also abundant in tocopherols (99.4 mg/100 g), with the presence of γ- (ca. 54%), δ- (ca. 31%) and α- (ca. 15%) homologues. The least rich and varied was pork lard, in which only the presence of α-tocopherol in the amount of 3.1 mg/100 g was determined.

### Fatty Acid Composition of Adipose Tissue and Liver in Rats

The fatty acid composition of animal tissue lipids is predominantly determined by dietary intake and endogenous synthesis [24]. The results of the present study indicate that the fatty acid profile of adipose tissue and liver was changed under the impact of different types of diet (Table 3). Independently of the used diet the adipose tissue was composed of four main fatty acids: palmitic, oleic, linoleic and palmitoleic, and the total share of these acids was close to 87% for rats feed with lard and sea-buckthorn oil and slightly higher (ca. 90%) for soybean oil diet. Saturated acids such as myristic, pentadecanoic and stearic constituted from 6 to 9% of the total. Small amounts of vaccenic and α-linolenic acids and traces of ARA and DHA acids were also found.

**Table 3:** Fatty acids composition in experimental animals’ tissues (%)

	Pork lard	Soybean oil	Sea-buckthorn oil
Adipose tissue			
myristic C_14:0_	2.58 ± 0.97^A^	1.93 ± 0.16^A^	2.25 ± 0.40^A^
pentadecanoic C_15:0_	0.37 ± 0.90^A^	0.40 ± 0.61^A^	0.13 ± 0.36^A^
palmitic C_16:0_	30.47 ± 1.79^A^	24.34 ± 1.54^B^	29.51 ± 2.28^A^
palmitoleic C_16:1 ω7_	7.93 ± 0.96^A^	5.72 ± 0.91^B^	15.16 ± 1.40^C^
stearic C_18:0_	6.21 ± 0.59^A^	4.15 ± 0.34^B^	3.91 ± 0.67^B^
oleic C_18:1 ω9_	38.61 ± 2.15^A^	29.20 ± 2.29^B^	23.15 ± 0.87^C^
vaccenic C_18:1 ω7_	3.03 ± 1.88^A^	3.10 ± 1.30^A^	6.53 ± 1.18^B^
linoleic C_18:2 ω6_	10.62 ± 2.86^A^	30.20 ± 3.54^B^	19.37 ± 2.98^C^
α-linolenic C_18:3 ω3_	0.19 ± 0.37^A^	0.95 ± 0.76^B^	0.00 ± 0.00^A^
arachidonic C_20:4 ω6_	trace	trace	trace
docosahexaenoic C_22:6 ω3_	trace	trace	trace
SFA	39.63	30.82	35.8
MUFA	49.57	38.02	44.84
PUFA	10.81	31.15	19.37
ω3/ω6 ratio	0.02	0.03	-
Liver tissue			
miristic C_14:0_	1.31 ± 0.29^A^	1.18 ± 0.32^A^	0.98 ± 0.38^A^
pentadecanoic C_15:0_	0.35 ± 0.10^A^	0.52 ± 0.34^A^	0.44 ± 0.17^A^
palmitic C_16:0_	21.11 ± 3.32^A^	18.35 ± 3.15^A^	19.89 ± 1.99^A^
palmitoleic C_16:1 ω7_	13.07 ± 2.21^A^	8.94 ± 2.66^B^	19.30 ± 1.96^C^
stearic C_18:0_	3.51 ± 0.56^A^	2.69 ± 1.04^B^	2.22 ± 0.61^B^
oleic C_18:1ω9_	42.45 ± 2.74^A^	28.75 ± 1.62^B^	23.85 ± 0.99^C^
vaccenic C_18:1 ω7_	6.05 ± 0.44^A^	4.12 ± 0.82^B^	7.92 ± 0.80^C^
linoleic C_18:2 ω6_	5.62 ± 2.07^A^	27.85 ± 8.00^B^	18.22 ± 2.74^C^
α-linolenic C_18:3 ω3_	0.19 ± 0.09^A^	0.91 ± 0.42^B^	0.64 ± 0.16^B^
arachidonic C_20:4 ω6_	0.29 ± 0.11^AB^	0.31 ± 0.74^A^	0.17 ± 0.09^B^
docosahexaenoic C_22:6 ω3_	1.99 ± 1.13^A^	1.55 ± 0.90^A^	1.73 ± 0.79^A^
other	4.07 ± 0.73^A^	4.82 ± 1.48^A^	4.63 ± 0.80^A^
SFA	26.28	22.74	23.53
MUFA	61.57	41.81	51.07
PUFA	8.09	30.62	20.76
ω3/ ω6 ratio	0.37	0.09	0.13

Values within a row with different letters (A,B,C…) are significantly different (*p* = 0.05)

Although the total share of main fatty acids in adipose tissue appears to have been conserved, a high variation of individual acids was observed. It generally reflected the composition of fat in the diets, since, in all cases, the predominant fatty acids of the meal were also predominant in adipose tissue. It was found that feeding with lard resulted in the highest share of oleic and palmitic acids, with soybean oil in linoleic and oleic acids and with sea-buckthorn in palmitic, palmitoleic and oleic acids. This confirms that adipose tissue is the best indicator of the long-term dietary intake of fats [24]. The observed correlation between diet and composition of adipose tissue is important, since the texture of human storage fatty tissue (softness, rigidness, compactness) may be modified by the different composition of fats in a diet. It has been noted that a diet with sea-buckthorn oil caused a significant increase in the share of palmitoleic and vaccenic acids, accompanied by a simultaneous decrease of stearic and oleic acids. A similar high sensitivity of the fatty acid composition of adipose tissue to the type of diet was recently demonstrated by Chandrashekar et al. [25]. In experiments using coconut, soybean and sunflower oils they found that the share of administered fatty acids is reflected in the profile of fatty acids of this storage tissue, with the highest sensitivity of linoleic acid.

It was previously found that the fatty acid profile of liver differs from that of adipose tissue [25, 26]. Liver generally contains a lower share of oleic acid, but contains considerable amounts of long-chain PUFAs, such as ARA and DHA [25, 27]. The relative ratio of PUFA/SFA varies between 0.5–1.5 in regard to the type of fat in the diet [25]. In our study, this ratio varied from 0.31 for lard to 1.34 for soybean oil (Table 3), but we suppose that it was slightly underestimated since we only analysed the neutral fat, which was hexane-extracted. The rat livers in our study had the highest share of oleic (23.8–42.4% in relation to diet), palmitic (18.3–21.1%) and linoleic (5.6–27.8%) acids. These ranges are close to previous findings [25]. In contrast to the cited work, we found a high share of palmitoleic acid (8.9–19.3% in relation to the diet), while the share of long-chain PUFAs was substantially lower. Especially noteworthy was the high share of palmitoleic acid (19.3%) as a result of feeding with sea-buckthorn oil (a similar phenomenon as observed in adipose tissue). However, a similar effect was noted for lard containing only ca. 3% of palmitoleic acid. It shows that palmitoleic acid is preferentially deposited in the liver. There was a ca. 116% increase of this acid observed in the liver after the consumption of sea-buckthorn pulp oil and ca. 46% increase after feeding with lard (in comparison to soybean oil – free of palmitoleic acid). This may have significant health consequences, since palmitoleic acid acts as a lipokine hormone and affects insulin sensitivity and fat accumulation in the liver [28].

### Retinol, Its Esters and α-Tocopherol in Liver

Dietary carotenoids and tocopherols, as other lipophilic components, are absorbed in the intestine via passive diffusion or via scavenger receptor class-B type I proteins after being incorporated into the micelles [15, 29, 30]. Inside the mucosal cells, they are incorporated into the chylomicrons and then released to the lymph. Chylomicrons are then catabolized to their remnants, incorporated into the lipoproteins at the site of the liver and then released into the blood stream [30]. Carotenoids and tocopherols are absorbed differentially by various tissues, with the predominant intake of carotenoids by liver and adipose tissue [29, 31], whereas tocopherols by liver, lung, brain and reproductive tissues [15]. Current data show that the conversion efficiency of food β-carotene into retinol (vitamin A) in humans ranges from 3.6:1 to 28:1 by weight [more details in 32] . Bioconversion is highly dependent on carotenoid structure, particularly on the most active *all*-*trans* β-isomer form [32]. Generally, created vitamin A can be stored in animal tissues very effectively [33]. In the case of tocopherols, the main form retained in human plasma is RRR-α-tocopherol [6, 10]. Generally, tocopherol bioavailability, metabolism into vitamin E and excretion in faeces, bile and urine are tightly regulated, which prevents toxic accumulation even at high-dose supplementation [6].

The present study found the highest level of retinol and its esters in livers of rats fed a diet containing sea-buckthorn oil (Table 4). The content of the substances under study in this sample was: 553.4 nmol/g for retinol and 190.8 nmol/g for its esters. The content of these compounds in a diet with soybean oil was lower by nearly 28% for retinol and 6% for its esters. The smallest content of retinol (293.8 nmol/g) and its esters (147.1 nmol/g) was found in the liver of rats fed a pork lard diet.

**Table 4:** Retinol, retinol esters and α-tocopherol in experimental animals’ liver tissues

	Retinol	Retinol esters	α-tocopherol
	nmol/g		
Pork lard	293.85 ± 93.76^A^	147.08 ± 49.95^A^	77.60 ± 28.87^A^
Soybean oil	397.25 ± 78.09^A^	179.93 ± 23.19^A^	104.05 ± 50.78^A^
Sea-buckthorn oil	553.43 ± 167.04^B^	190.78 ± 63.72^A^	104.46 ± 18.99^A^

Values within a column with different letters (A,B …) are significantly different (*p* = 0.05)

In total, the presence of sea-buckthorn oil in diet, in relation to feeding with pork lard, resulted in ca. 40% increase in liver saturation with vitamin A-like compounds. Guliyev et al. [7] confirmed the beneficial effect of sea-buckthorn oil in the treatment and protection of the liver. The cited authors stated that this oil considerably inhibited the formation of malondialdehyde in livers exposed to CCl_4_, ethanol, and paracetamol. Sea-buckthorn oil also decreased the level of alanine transaminase induced by CCl_4_ and paracetamol and prevented exhaustion of glutation in livers damaged by acetaminophen.

Although sea-buckthorn oil was the richest source of tocopherols, with α-tocopherol being the dominant homologue, the content of α-tocopherol in the analysed livers was similar in all feeding options (Table 4). It varied from 77.6 nmol/g for pork lard to ca. 104 nmol/g for both oils. These values are significantly higher than the data obtained by Jurczuk et al. [8], who determined the concentration of vitamin E in rat livers as being close to 20 μg/g of tissue. The important observation of our study was that the lowest coefficient of variation (18.2%) of this compound was found in the group of rats fed sea-buckthorn oil (for comparison, its value for soybean oil was 48.8% and for lard 37.2%). This may show that with a sea-buckthorn oil diet there was a saturation of liver with this compound. Asadian et al. [34] studied the degree of α-tocopherol absorption in the liver of sheep fed with its addition and found no significant increase in the liver under supplementation.

## Conclusions

It was found that the fatty acid profile of sources of fat in a diet was reflected in the composition of the adipose tissue and liver of rats. The addition of sea-buckthorn oil to fodder significantly increased the share of palmitoleic and vaccenic acids and simultaneously decreased the share of stearic and oleic acids in both analysed tissues. This oil was also a rich source of carotenoids and tocopherols in the diet, especially β-carotene and α-tocopherol, the homologues with the highest biological values. The delivered carotenoids were bio-converted into retinol and its esters and they increased the total concentration of these compounds in the liver**.** Despite the highest content of α-tocopherol, sea-buckthorn oil did not have a significant effect on its medium level in rat livers because of high individual variability of biological response between used animals.
